# Mavacamten rescues increased myofilament calcium sensitivity and dysregulation of Ca^2+^ flux caused by thin filament hypertrophic cardiomyopathy mutations

**DOI:** 10.1152/ajpheart.00023.2020

**Published:** 2020-02-21

**Authors:** Alexander J. Sparrow, Hugh Watkins, Matthew J. Daniels, Charles Redwood, Paul Robinson

**Affiliations:** ^1^Division of Cardiovascular Medicine, Radcliffe Department of Medicine, University of Oxford, Oxford, United Kingdom; ^2^British Heart Foundation Centre of Research Excellence, University of Oxford, Oxford, United Kingdom; ^3^Division of Cardiovascular Sciences, University of Manchester, Manchester, United Kingdom

**Keywords:** calcium, cardiomyocyte, hypertrophic cardiomyopathy, mavacamten, mutation

## Abstract

Thin filament hypertrophic cardiomyopathy (HCM) mutations increase myofilament Ca^2+^ sensitivity and alter Ca^2+^ handling and buffering. The myosin inhibitor mavacamten reverses the increased contractility caused by HCM thick filament mutations, and we here test its effect on HCM thin filament mutations where the mode of action is not known. Mavacamten (250 nM) partially reversed the increased Ca^2+^ sensitivity caused by HCM mutations Cardiac troponin T (cTnT) R92Q, and cardiac troponin I (cTnI) R145G in in vitro ATPase assays. The effect of mavacamten was also analyzed in cardiomyocyte models of cTnT R92Q and cTnI R145G containing cytoplasmic and myofilament specific Ca^2+^ sensors. While mavacamten rescued the hypercontracted basal sarcomere length, the reduced fractional shortening did not improve with mavacamten. Both mutations caused an increase in peak systolic Ca^2+^ detected at the myofilament, and this was completely rescued by 250 nM mavacamten. Systolic Ca^2+^ detected by the cytoplasmic sensor was also reduced by mavacamten treatment, although only R145G increased cytoplasmic Ca^2+^. There was also a reversal of Ca^2+^ decay time prolongation caused by both mutations at the myofilament but not in the cytoplasm. We thus show that mavacamten reverses some of the Ca^2+^-sensitive molecular and cellular changes caused by the HCM mutations, particularly altered Ca^2+^ flux at the myofilament. The reduction of peak systolic Ca^2+^ as a consequence of mavacamten treatment represents a novel mechanism by which the compound is able to reduce contractility, working synergistically with its direct effect on the myosin motor.

**NEW & NOTEWORTHY** Mavacamten, a myosin inhibitor, is currently in phase-3 clinical trials as a pharmacotherapy for hypertrophic cardiomyopathy (HCM). Its efficacy in HCM caused by mutations in thin filament proteins is not known. We show in reductionist and cellular models that mavacamten can rescue the effects of thin filament mutations on calcium sensitivity and calcium handling although it only partially rescues the contractile cellular phenotype and, in some cases, exacerbates the effect of the mutation.

## INTRODUCTION

Hypertrophic cardiomyopathy (HCM) is the most common inherited cardiac disease, globally affecting 1 in 500 people (10). It is predominantly caused by mutations in sarcomeric proteins (20, 21), which alter Ca^2+^ cycling, contractility (8, 13), and myocardial energetics (21). To date there is no widely available effective treatment that acts on the primary cause of the disease, the sarcomere. Current treatments include β-blockers that prevent arrhythmias (18) or Ca^2+^ channel blockers (2) such as verapamil or diltiazem, which act to prevent diastolic dysfunction by prolonging left ventricular filling time (18); however, they do not affect the underlying altered myofilament function (5).

To directly target the sarcomere, the myosin inhibitor, mavacamten (6) (or MYK-461), has been developed and is currently in clinical trials. Mavacamten has been shown to decrease the enhanced contractility caused by β-myosin heavy chain and myosin binding protein C mutations in vitro and to suppress HCM in a myosin mouse model (6, 9, 19); it has also been shown to affect the biochemical equilibrium of thick filaments during contractile activation to favor the “super-relaxed state” (1, 9). However, the efficacy of mavacamten in reversing the effect of mutations in thin filament proteins has not been reported. We have recently developed novel Ca^2+^ probes that report relative [Ca^2+^] in distinct cytoplasmic and myofilament-specific pools (17); using these in wild-type adult left ventricular cardiomyocytes, we have shown that mavacamten decreases the [Ca^2+^] in the cytoplasm and at the myofilament in addition to increasing the Ca^2+^ release rate from the myofilament. This suggests that mavacamten might qualitatively reverse the altered Ca^2+^ handling observed in thin filament Ca^2+^-sensitizing mutations (13). This study aims to assess this in a pairwise manner for the first time by testing the direct effect of mavacamten on HCM mutations [cardiac troponin T (cTnT) R92Q, and cardiac troponin I (cTnI) R145G] in the reductionist in vitro actomyosin ATPase assay and in an appropriate cellular model of transduced guinea pig cardiomyocytes (17). For both mutations, mavacamten elicited rescue of myofilament Ca^2+^ sensitivity changes and partial rescue of the Ca^2+^ phenotype at the myofilament.

## METHODS

### 

#### Protein purification.

Wild-type and mutant human recombinant cTnT, cTnT R92Q, cTnI, cTnI R145G, troponin C (TnC), and Ala-Ser-α-tropomyosin were purified as previously described (4, 14). Troponin complex containing either wild-type (WT) cTnT, cTnI, TnC, or cTnT R92Q, WT cTnI, TnC, or WT cTnT, cTnI R145G, and TnC were reconstituted by stepwise dialysis as previously described (4). Urea was first reduced from 6 to 0 M and then KCl from 1 M to 200 mM in five sequential dialyses. Troponin complex was separated from unincorporated subunits using size exclusion chromatography, and purity was analyzed by SDS-PAGE. Purified troponin complexes were dialyzed into ATPase buffer, consisting of (in mM) 5 1,4-piperazinediethanesulphate (pH 7.0), 3.87 MgCl_2_, and 1 DTT, for in vitro actomyosin ATPase experiments. Actin and myosin were extracted from rabbit skeletal muscle, and myosin subfragment-1 (S-1) was prepared by limited proteolysis as previously described (11, 22).

#### In vitro actomyosin ATPase assays.

ATPase assays were carried out as previously described (12, 14). The components (in μM) 3.5 actin, 0.5 myosin S-1, 0.5 Ala-Ser-α-tropomyosin, and 0.5 troponin complex were mixed in ATPase buffer and were aliquoted and set to a range of [Ca^2+^]_free_ from pCa 4.5 to 8.5. ATPase reactions were started by addition of 3 mM ATP, incubated at 37°C for 7 min, and quenched in 5% TCA, 1% ammonium molybdate in 0.5 M H_2_SO_4_, followed by 40% iron(II)sulfate in 0.5 M H_2_SO_4_ was used to measure inorganic phosphate. Absorbance (A_700_) measurements were converted to absolute activity (s^−1^), and with the use of KaleidaGraph (Synergy Software), calcium-sensitivity data was fitted to the Hill equation, as follows:

$$\text{A}={\text{A}}_{\text{min}}+\left[\frac{\left({\text{A}}_{\text{max}}-{\text{A}}_{\text{min}}\right)}{1+10^{\left(\text{pCa}-{\text{pCa}}_{50}\right)\times n_{\text{H}}}}\right]$$

where A = ATPase rate, A_min_ = minimum ATPase rate, A_max_ = maximum ATPase rate, pCa = −log [Ca^2+^], pCa_50_ = −log [Ca^2+^] required for half maximum ATPase activity, and *n*_H_ = Hill coefficient.

#### Cardiomyocyte isolation.

This investigation was approved by the Animal Welfare and Ethical Review Board at the University of Oxford and conforms to the UK Animals (Scientific Procedures) Act, 1986, incorporating Directive 2010/63/EU of the European Parliament. As previously described (13), briefly, adult left ventricular cardiomyocytes were isolated from guinea pig (male, 400 g) hearts using our standard Langendorff procedure. Left ventricular cardiomyocytes (1.5 × 10^5^ cells per ml) were incubated in ACCITT_3_ culture medium at 37°C and 5% CO_2_.

#### Adenoviral transduction.

Cardiomyocytes were adenovirally cotransduced for 48 h with either red-fluorescent, genetically encoded Ca^2+^ indicator for optical imaging (RGECO), RGECO-TnI, or RGECO-TnT and either WT cTnT, cTnT R92Q, WT cTnI, or cTnI R145G to give expression the WT and mutant troponins at an approximate 50% level compared with endogenous troponin as previously implemented and described (13, 17).

#### Measurement of sarcomere shortening.

Sarcomere shortening measurements were performed using IonOptix μstep apparatus. Cultured cardiomyocytes were perfused with perfusion buffer, consisting of (in mM)150 NaCl, 5 HEPES, 5.5 glucose, 1 MgCl_2_, 1.8 KCl, 0.35 NaH_2_PO_3_, and 1.8 CaCl_2_ pH 7.4) and electrically paced at 40 V, 0.5 Hz, and at 37°C. Sarcomere shortening was captured by Fourier transform of the cardiomyocyte striations under phase contrast microscopy at 250 Hz. All contracting cardiomyocytes were measured.

#### Calcium imaging.

Cardiomyocytes from each adenovirally cotransduced group were split in two (DMSO control and mavacamten treatment). After 15 min incubation at 37°C, cardiomyocytes were imaged on an Olympus IX81 inverted microscope (Olympus, Japan) with a C-9100-13 EMCCD camera (Hamamatsu, Japan). Videos of 0.5-Hz electrically paced cardiomyocytes at 37°C were acquired at 25 frames/s (560/25 nm excitation, 620/60 nm emission with a 565 nm dichroic mirror). Raw image data were extracted using xcellence (Olympus), analyzed in Excel (Microsoft) and Ca^2+^ transient parameters extracted; all baseline variation of Ca^2+^ transients derived from differential expression of RGECO or driven by altered basal sarcomere lengths (via myofilament Ca^2+^ buffering) were normalized by calculating ΔF/F for each transient (17).

#### Statistics.

ATPase groups were tested for normality (D’Agostino and Pearson test), and either an unpaired *t*-test or Mann-Whitney test was performed (GraphPad Prism). All cardiomyocyte comparisons were from at least three separate cell isolations with a similar number of cells from each isolation analyzed. Furthermore, all mutant and/or treatment groups were compared with wild type by splitting the same cell isolation to create pairwise comparisons and reduce variability. Any cell with extracted parameters of baseline, amplitude, or kinetics exceeding two standard deviations from the mean upon analysis of both sarcomere shortening and Ca^2+^ transients were excluded due to phenotypic heterogeneity arising in cultured primary cells. Groups were tested for normality (D’Agostino and Pearson test) and either an ordinary one-way ANOVA with Tukey’s or Kruskal-Wallis test with Dunn’s test was performed (GraphPad Prism).

## RESULTS

**Fig. 1.**
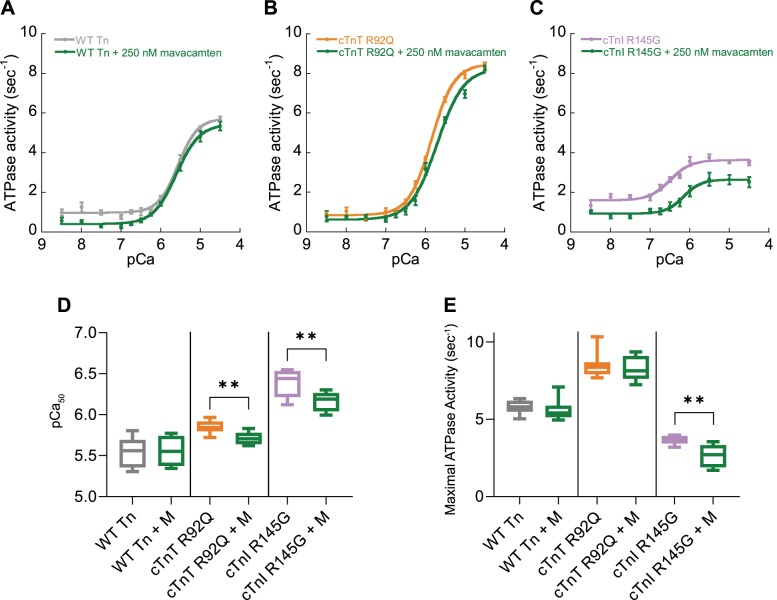
The effect of mavacamten on the myofilament ATPase. Myofilament function was assessed using in vitro actin activated actomyosin S1 ATPase assays. Myofilaments containing wild-type (WT) troponin (Tn) complexes (gray; *A*, *D*, and *E*), troponin complex reconstituted containing cTnT R92Q subunits (orange; *B*, *D*, and *E*), or cTnI R145G (purple; *C*, *D*, and *E*) were compared with those treated with mavacamten (M, green; *A–E*); *n* = 8, means ± SE. −Log [Ca^2+^] required for half maximum ATPase activity (pCa_50_; *D*) and maximal ATPase activity (*E*) are plotted comparing either wild-type troponin, troponin containing troponin T (TnT) R92Q, or cardiac troponin I (cTnI) R145G subunits with those treated with 250 nM mavacamten. Box and whisker plots (*D* and *E*) give the median average, interquartile range (box), and minimum and maximum data spread (whiskers). **P < 0.01, using Student’s *t*-test (*D*) or Mann-Whitney test (*E*) comparing mutant troponin to mavacamten-treated mutant troponin.

To assess the efficacy of mavacamten on the Ca^2+^ regulation of actomyosin ATPase activity, we measured in vitro skeletal muscle myosin subfragment-1 (S-1) ATPase activity activated by reconstituted thin filaments containing either WT recombinant human troponin subunits or those containing the HCM causing mutations cTnT R92Q or cTnI R145G (Fig. 1). Both mutant troponins regulated ATPase activity with lower pCa_50_ in the presence of 250 nM mavacamten (Fig. 1, *B*–*E*), despite the reported lower *K*_d_ of mavacamten for skeletal muscle myosin (4.7 μM vs. 0.3 μM) for cardiac myosin (6) (Fig. 1, *A*, *D*, and *E*). We have previously described the relationship between alterations to myofilament Ca^2+^ affinity and cardiomyocyte Ca^2+^ handling via altered myofilament Ca^2+^ buffering (13). Furthermore, we have observed increased systolic [Ca^2+^] with these HCM mutations (17) and now aim to test whether the potent myofilament Ca^2+^ desensitization of mavacamten can also rescue this feature of the disease molecular phenotype. We therefore assessed the effect of mavacamten in isolated adult guinea pig left ventricular cardiomyocytes (GPCMs) transfected by adenoviruses to express cTnT R92Q or cTnI R145G to model autosomal dominant human HCM (replacement level of ~54% for cTnT R92Q and ~49% for cTnI R145 (13). We also expressed either cytoplasmic RGECO, or myofilament specific RGECO-TnT by adenoviral transduction to observe the whole cell or myofilament localized Ca^2+^ changes (17). Specific multiplicities of infection, as previously described, were used to ensure consistent expression of the Ca^2+^ sensors and either cTnT R92Q or cTnI R145G (17).

**Fig. 2.**
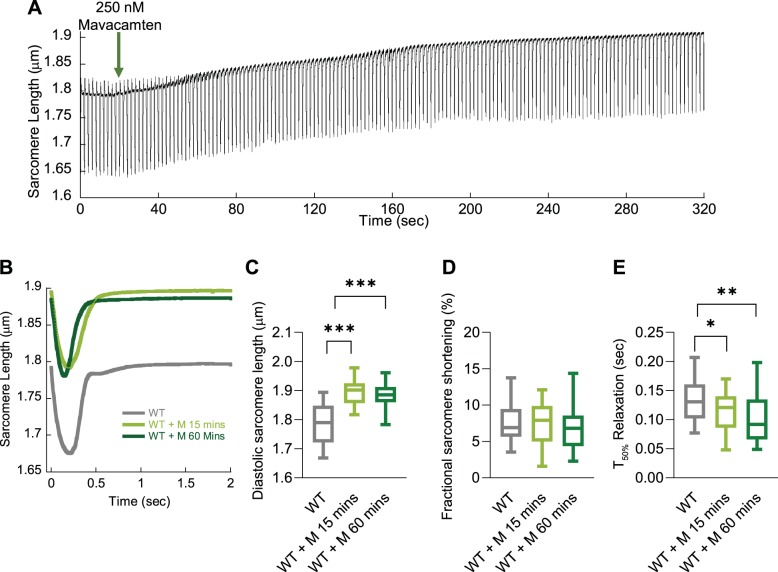
The effect of mavacamten on unloaded sarcomere shortening over time. Representative raw sarcomere length trace of a guinea pig left ventricular cardiomyocyte (GPCM) trace to show the real-time change to basal sarcomere length over time after addition of 250 nM mavacamten (green arrow; *A*). Averaged sarcomere length traces of wild-type (WT) control GPCMs ± mavacamten incubated for either 15 or 60 min (*B*). Diastolic sarcomere length (*C*), fractional shortening (*D*), and time to 50% (*T*_50_) relaxation (*E*) are plotted for WT, 250 nM mavacamten (M) for 15 min, and mavacamten (M) for 60 min; *n* = 30–41 cells from *n* = 4 isolations (*B–E*). Box and whisker plots (*C–E*) give the median average, interquartile range (box), and minimum and maximum data spread (whiskers). *P < 0.05, **P < 0.01, and ***P < 0.001.

We found that the direct administration of 250 nM mavacamten to wild-type GPCMs by perfusion switching (Fig. 2*A*) causes an alteration of basal sarcomere length of ~0.1 μm following 3 to 4 min of perfusion. This appears to be the maximal change to basal sarcomere length, as preincubation of 250 nM for both 15 min or 1 h does not significantly increase this parameter (Fig. 2, *B* and *C*). Interestingly, we find that fractional shortening is unchanged and speed of relaxation is increased at both time points (Fig. 2, *D* and *E*). We therefore chose a 15-min incubation with 250 nM mavacamten for all subsequent GPCM experiments; this reflects the optimal temporal effect of the drug and concentrations close to the *K*_d_ for cardiac β-myosin (6, 7) and 250 nM mavacamten was used in the original paper as a working concentration for drug dosing of myosin R403Q HCM transgenic mice (6).

**Fig. 3.**
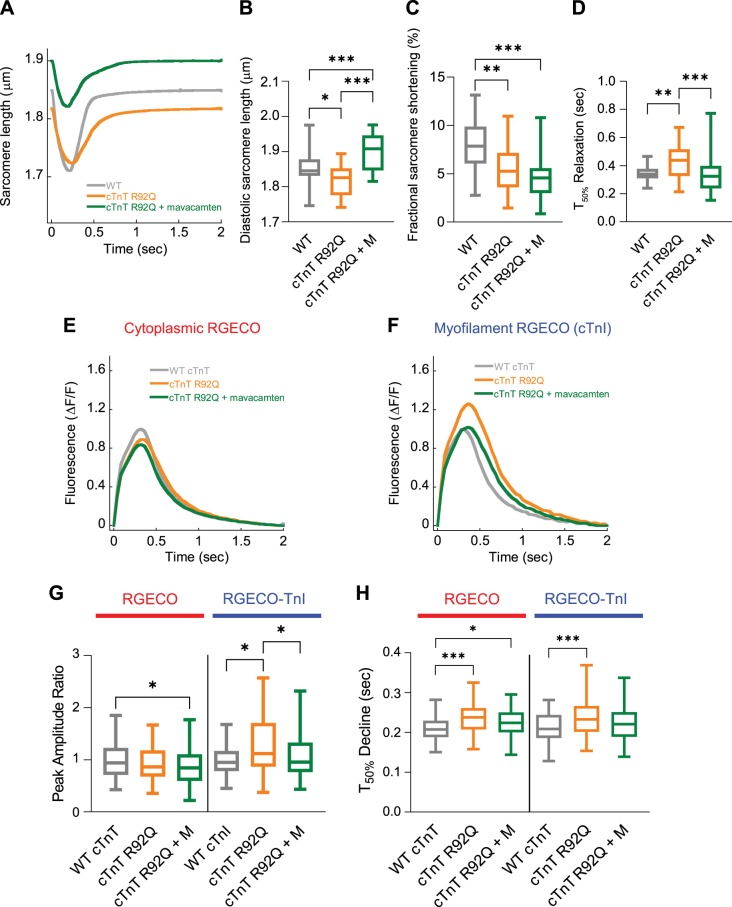
The effect of mavacamten on contractility and cytoplasmic/myofilament localized Ca^2+^ transients with adenovirally transduced cardiac troponin T (cTnT) R92Q. Averaged sarcomere length traces of adult guinea pig left ventricular cardiomyocytes (GPCMs) transduced with either wild-type (WT) cTnT or cTnT R92Q ± mavacamten (*A*). Diastolic sarcomere length (*B*), fractional shortening (*C*), and time to 50% (*T*_50_) relaxation (*D*) are plotted for WT, mutant troponin, and mutant troponin ± 250 nM mavacamten (M). Averaged Ca^2+^ transients GPCMs transduced with red-fluorescent, genetically encoded Ca^2+^ indicator for optical imaging (RGECO; *E*) or RGECO-TnI (*F*) and either WT cTnT or cTnT R92Q ± 250 nM mavacamten. Peak amplitude ratios (*G*) and time to 50% decline (*H*) are plotted for WT, mutant troponin, and mutant troponin + 250 nM mavacamten (M); *n* = 30–41 cells from *n* = 4 isolations (*A–D*) or *n* = 65–86 cells from *n* = 3 isolations (*E–H*). Box and whisker plots (*B–D*, *G*, and *H*) give the median average, interquartile range (box), and minimum and maximum data spread (whiskers). *P < 0.05, **P < 0.01, and ***P < 0.001.

**Fig. 4.**
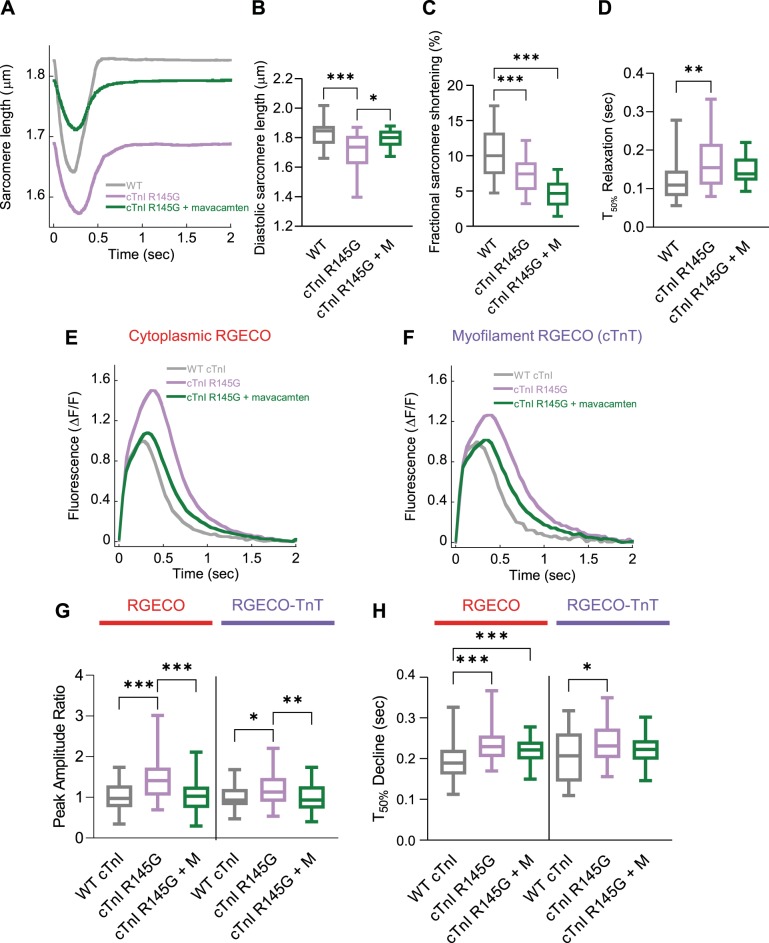
The effect of mavacamten on contractility and cytoplasmic/myofilament localized Ca^2+^ transients with adenovirally transduced cardiac troponin I (cTnI) R145G. Averaged sarcomere length traces of adult guinea pig left ventricular cardiomyocytes (GPCMs) transduced with either wild-type (WT) cTnI or cTnI R145G ± mavacamten (*A*). Diastolic sarcomere length (*B*), fractional shortening (*C*), and time to 50% (*T*_50_) relaxation (*D*) are plotted for WT, mutant troponin, and mutant troponin ± 250 nM mavacamten (M). Averaged Ca^2+^ transients GPCMs transduced with red-fluorescent, genetically encoded Ca^2+^ indicator for optical imaging (RGECO; *E*) or RGECO-TnT (*F*) and either WT cTnI or cTnI R145G ± 250 nM mavacamten. Peak amplitude ratios (*G*) and time to 50% decline (*H*) are plotted for WT, mutant troponin, and mutant troponin + 250 nM mavacamten (M); *n* = 12–32 (*A–D*) or *n* = 38–71 (*E–H*) cells from *n* = 3 isolations. Box and whisker plots (*B–D*, *G*, and *H*) give the median average, interquartile range (box), and minimum and maximum data spread (whiskers). *P < 0.05, **P < 0.01, and ***P < 0.001.

Using the conditions above, we assessed the effect of mavacamten on the function of GPCMs expressing HCM causing mutations. We observed relaxation of the hypercontracted diastolic sarcomere length beyond the wild-type length. With respect to sarcomere length, wild-type cells measured 1.85 ± 0.01 µm, cells expressing cTnT R92Q measured 1.82 ± 0.01 µm (*P* < 0.05), and cTnT R92Q cells treated with mavacamten measured 1.90 ± 0.01 µm (*P* < 0.001) (Fig. 3, *A* and *B*). However, we observed rescue of hypercontracted diastolic sarcomere length in cells expressing cTnI R145G, shifting from 1.71 ± 0.02 to 1.80 ± 0.02 μm (*P* < 0.001) with wild type measured at 1.82 ± 0.02 μm (*P* > 0.05) (Fig. 4, *A* and *B*).

Mavacamten did not reverse and in fact exacerbated the reduced fractional shortening in both cTnT R92Q and cTnI R145G cardiomyocytes. Control cardiomyocytes had 7.90 ± 0.39% fractional shortening while cTnT R92Q cells had 5.64 ± 0.45% (*P* < 0.01), and cTnT R92Q cells with mavacamten had 4.72 ± 0.43% (*P* < 0.001) fractional shortening (Fig. 3*C*). Mavacamten had a similar effect on cTnI R145G cells: fractional shortening was decreased (nonsignificant; *P* = 0.0591) from 7.17 ± 0.42 (*P* < 0.001 c.f. control) to 4.76 ± 0.58% (*P* < 0.001; c.f. control) compared with 10.59 ± 0.72%, for wild type (Fig. 4*C*). The increase observed in time to 50% relaxation with cTnT R92Q and cTnI R145G was reversed upon treatment with mavacamten (Figs. 3*D* and 4*D*).

In GPCMs expressing cTnT R92Q, peak systolic fluorescence was decreased in cells treated with mavacamten compared with wild type when observed with the cytoplasmic Ca^2+^ sensor; however, the increase observed in peak systolic fluorescence when measured using the myofilament Ca^2+^ sensor was reversed with 250 nM mavacamten (Fig. 3, *E*–*G*). In GPCMs expressing cTnI R145G, we observed complete rescue of increased systolic Ca^2+^ upon treatment with 250 nM mavacamten using both the cytosolic- and myofilament-localized sensors (Fig. 4, *E*–*G*).

The T_50_ Ca^2+^ transient decline remained prolonged in the cytosol in cTnT R92Q and cTnI R145G with mavacamten; however, T_50_ decline at the myofilament was reversed to control levels with 250 nM mavacamten (Figs. 3*H* and 4*H*) in both mutations. Thus, if only a cytoplasmic sensor had been used, no rescue of this parameter would have been observed with mavacamten in either cTnT R92Q- or cTnI R145G-expressing cells, underlining the need for sensors that are localized at the site of the mutation and at the primary mechanistic location of increased myofilament sensitivity.

## DISCUSSION

Mavacamten (250 nM) reduced Ca^2+^ sensitivity of in vitro actomyosin ATPase regulation by both cTnT R92Q or cTnI R145G HCM mutants but did not affect regulation by wild-type troponin, the latter in excellent agreement with Kawas et al. (7) who similarly used rabbit skeletal myosin S-1. Using intact wild-type cardiomyocytes, we have previously shown a decrease in systolic [Ca^2+^] both in the cytoplasm and at the myofilament (17). This effect might be due to mavacamten stabilizing the super-relaxed state of myosin (1, 15), which is not present in myosin S-1 used in the in vitro assays or the different *K*_d_ of mavacamten binding to skeletal verses myosin isoforms. Thus, while ATPase assays can be a useful screening tool, they do not recapitulate the full range of myosin interactions in the intact sarcomere. This could also explain the variable effect of mavacamten in reducing maximal ATPase activity versus the observed reduction of maximal force in intact muscle fiber assays (1, 6, 9, 15).

The complete reversal of the systolic fluorescence increase by mavacamten is interesting since it implies that the effects of the drug on contractility are mediated not only by effects at the level of the myosin motor but, additionally and synergistically, by reducing systolic Ca^2+^ to lower thin filament activation. However, in the case of the cTnT R92Q mutant, cytoplasmic Ca^2+^ levels were reduced to below wild-type levels upon mavacamten treatment of mutant cells. This suggests that the genotype of patients may need to be carefully considered in translational studies. These data also suggest that mavacamten can partially rescue the primary disease driver of increased Ca^2+^ sensitivity in thin filament mutations, and thus mavacamten might also be clinically effective for thin filament Ca^2+^-sensitizing mutations in addition to the thick filament mutations that enhance myosin activation and destabilize the myosin super-relaxed state. However, we cannot exclude the possibility that mavacamten could also have novel off-target effects on the Ca^2+^-handling machinery of the cardiomyocyte in addition to modulating myofilament Ca^2+^ sensitivity.

While mavacamten rescues the hypercontracted basal sarcomere length observed in cells with the cTnI R145G mutation, it pushed the basal sarcomere length beyond control length to a more relaxed state in cTnT R92Q cells. Mavacamten also does not rescue the decreased fractional shortening in cTnT R92Q or cTnI R145G and indeed further decreases it, thus suggesting that while mavacamten rescues some aspects of thin filament HCM pathophysiology, it may exacerbate others, which may not even be addressed by titrations of drug concentration. Caution should be advised and the precise molecular mechanisms of disease pathogenesis considered when interpreting the potential benefits of mavacamten for mutations of the thin filament. For example, TnI R145G directly alters the Ca^2+^ binding of TnC (3, 12), thereby directly affecting Ca^2+^ activated contractility, whereas the TnT R92Q mutation affects cooperative communication between troponin units on the thin filament that indirectly sensitizes myofilament Ca^2+^ binding (14). Similarly, it is well established that length-dependent activation can inherently alter the kinetics and force of contractility and that HCM mutations can impact this relationship (16). These phenomena may in part explain some of the differential functional effects of mavacamten in our models. Targeting Ca^2+^ sensitivity directly by modulating TnC might provide an alternative strategy to rectify the increase Ca^2+^ sensitivity observed in HCM without unnecessary drawbacks, such as overt contractile suppression observed with mavacamten (6).

## GRANTS

This work was funded by British Heart Foundation Grant PG/18/68/33883 and British Heart Foundation Centre of Research Excellence (Oxford) Grant RE/13/1/30181. A. J. Sparrow (PG/18/68/33883) and P. Robinson (CH/1992001/6764) are both supported by the British Heart Foundation. M. J. Daniels was funded by the Wellcome Trust Grant WT098519MA.

## DISCLOSURES

No conflicts of interest, financial or otherwise, are declared by the authors.

## AUTHOR CONTRIBUTIONS

A.J.S., M.J.D., C.R., and P.R. conceived and designed research; A.J.S. and P.R. performed experiments; A.J.S. and P.R. analyzed data; A.J.S., C.R., and P.R. interpreted results of experiments; A.J.S. and P.R. prepared figures; A.J.S. and C.R. drafted manuscript; A.J.S., H.W., M.J.D., C.R., and P.R. edited and revised manuscript; A.J.S., H.W., M.J.D., C.R., and P.R. approved final version of manuscript.
